# Point-of-Care Bilirubin Testing in Neonates: Comparative Performance of Blood Gas Analysis and Transcutaneous Bilirubinometry

**DOI:** 10.3390/healthcare14030370

**Published:** 2026-02-01

**Authors:** Andrew Xu, Bincy Francis, Kay Weng Choy, George Francis Dargaville, Amy Surkitt, David Tran, Rami Subhi, Wei Qi Fan

**Affiliations:** 1 Faculty of Medicine, Dentistry and Health Sciences, Melbourne University, Melbourne 3010, Australia; d.xu22@student.unimelb.edu.au (A.X.); david.tran2@nh.org.au (D.T.); rami.subhi3@nh.org.au (R.S.); 2 Northern Health, Melbourne 3076, Australiakayweng.choy@nh.org.au (K.W.C.);

**Keywords:** neonatal jaundice, hyperbilirubinaemia, transcutaneous bilirubinometry, blood gas analyser, point-of-care testing, serum bilirubin, diagnostic accuracy, phototherapy

## Abstract

**Highlights:**

**What are the main findings?**

- Blood gas analyser bilirubin showed stronger agreement with central laboratory serum bilirubin (R^2^ = 0.88) than transcutaneous bilirubinometry (R^2^ = 0.43), with blood gas analyser maintaining accuracy regardless of pre- vs. post-phototherapy or haemoglobin levels.
- Transcutaneous bilirubinometry accuracy significantly reduced post-phototherapy and showed reduced predictive value in darker-skinned neonates (Fitzpatrick III–VI), while the blood gas analyser demonstrated superior diagnostic performance with a diagnostic odds ratio of 47.5.

**What is the implication of the main findings?**

- Blood gas analysers represent a more reliable point-of-care alternative to serum bilirubin than transcutaneous bilirubinometry for neonatal hyperbilirubinaemia screening and hyperbilirubinaemia tracking, particularly valuable in time-critical or resource-limited clinical settings, where rapid, accurate results are essential.
- While transcutaneous bilirubinometry remains useful as a non-invasive screening tool, positive findings require confirmatory serum bilirubin testing, especially in post-phototherapy neonates and those with darker skin tones, to prevent misclassification and overtreatment.

**Abstract:**

**Background**: Neonatal jaundice is a common condition with potentially severe complications such as bilirubin-induced neurological dysfunction and kernicterus. While serum bilirubin (SBR) remains the standard laboratory measurement, point-of-care methods, such as transcutaneous bilirubinometry (TcB) and blood gas analysers (BGAs), offer rapid, less invasive alternatives. Direct comparisons of their diagnostic accuracy remain limited. **Objective**: The aim of this study was to assess and compare diagnostic accuracy and clinical utility of TcB and BGA against SBR in neonatal hyperbilirubinaemia screening. **Methods**: This retrospective study included neonates (n = 221) with concurrent SBR, BGA, and TcB measurements (n = 333). Assessment was via Passing–Bablok regression, Bland–Altman analysis, and Spearman correlation. Diagnostic performance was evaluated against jaundice thresholds in phototherapy charts (≥95th percentile threshold). Subgroup analyses considered phototherapy status, haemoglobin concentration, and Fitzpatrick skin type. **Results**: BGA showed stronger agreement with SBR (R^2^ = 0.88) than TcB (R^2^ = 0.43). BGA remained accurate regardless of phototherapy or haemoglobin levels. TcB accuracy declined post-phototherapy with reduced predictive value in darker-skinned neonates (Fitzpatrick III–VI) and increased false discovery rates. Both methods demonstrated low sensitivity (45.8%) but high specificity (>95%) and negative predictive value (~91%) for clinically significant hyperbilirubinaemia. BGA had a higher diagnostic odds ratio (47.5) than TcB (19.3). When individual patient sequential SBR and BGA measurements were compared for jaundice tracking (n = 175), there was high correlation, (r = 0.971) with no statistical differences, and 50% of measurements achieving agreement within 10 μmol/L. **Conclusions**: BGA is a more reliable alternative to SBR than TcB, particularly in time-critical or resource-limited settings. While TcB remains a non-invasive screening tool, limited accuracy post-phototherapy and with darker skinned neonates indicate confirmatory SBR testing. These findings support the selective and context-aware use of BGA and TcB to optimise neonatal hyperbilirubinaemia management and reduce interventions.

## 1. Introduction

Neonatal jaundice, clinically defined as hyperbilirubinaemia, is a common condition characterised by yellow discolouration of the skin and sclera due to elevated levels of bilirubin in the bloodstream [1]. Bilirubin is a byproduct of the normal breakdown of foetal haemoglobin, and its accumulation occurs when production exceeds the capacity of the immature neonatal liver to conjugate and excrete it [2]. The aetiology of neonatal jaundice is multifactorial; increased bilirubin production may occur due to enhanced haemolysis associated with Rhesus or ABO incompatibility, glucose-6-phosphate dehydrogenase deficiency, and other haemolytic disorders [3]. Additionally, prematurity contributes to decreased bilirubin clearance due to immature hepatic conjugation and reduced intestinal excretion. Other factors, including breastfeeding, bruising, and sepsis, can further exacerbate bilirubin accumulation. Whilst physiological neonatal jaundice is often transient and benign, severe hyperbilirubinaemia can lead to bilirubin-induced neurological dysfunction and kernicterus—conditions that may result in permanent neurological damage or death if not promptly recognised and managed [3].

Therefore, timely diagnosis of hyperbilirubinaemia and appropriate intervention are critical; however, the decision to initiate treatment depends on bilirubin levels that vary according to gestational age, birth weight, and other risk factors [4]. Phototherapy is the primary treatment modality via converting bilirubin into a water-soluble form that can be excreted, thereby reducing serum concentrations and preventing neurotoxicity [5]. In cases of severe hyperbilirubinaemia, exchange transfusion may also be required to rapidly decrease serum bilirubin concentration [5]. Consequently, the accuracy and speed of bilirubin measurement directly impact clinical outcomes.

Current diagnostic guidelines begin with clinical examination, where the visual assessment of skin and sclera is performed [5]. Neonates with a high index of suspicion then undergo objective measurement via laboratory determination of serum bilirubin (SBR) [1]. Whilst the most accurate methodology for bilirubin measurement is via high performance liquid chromatography, it is impractical in routine use [6]. Laboratory measurements are routinely performed on automated analysers with the photometric diazo methodology predominating, and results are typically interpreted in conjunction with risk stratification charts (such as the Bhutani nomogram) that plot bilirubin levels against postnatal age to guide treatment decisions [5]. The Diazo SBR method is considered relatively accurate but is subject to interference—especially from sample haemolysis [6]. Despite its laboratory practicality, the SBR assay has several clinical shortcomings: it requires invasive blood sampling and has an inherent processing delay, which may result in treatment delays in a time-critical neonatal setting.

In response to these limitations, recent technologies have been developed to facilitate rapid and less invasive screening for neonatal jaundice. Two such promising methods are transcutaneous bilirubinometry (TcB) [7] and whole-blood bilirubin measurements using blood gas analysers (BGA) [8]. TcB devices estimate bilirubin levels by measuring skin reflectance and provide immediate and painless results. Similarly, blood gas analysers have been adapted to measure blood bilirubin directly from capillary samples, offering the advantage of rapid results and requiring only minimal blood volumes compared to SBR. These point-of-care methods have been compared individually to serum bilirubin levels found in several previous studies [8,9,10,11,12,13,14] and systematic reviews [15,16], with findings generally supporting their utility as screening tools while also highlighting issues related to calibration, accuracy at high bilirubin concentrations, and influences from factors such as skin colour [17] and haemoglobin concentration [14].

Whilst prior research has evaluated TcB and BGA as point-of-care methods separately against SBR, to date, no study has directly compared both measures within the same study cohort to comprehensively assess their relative accuracy and clinical utility for diagnosing and managing neonatal jaundice. Such a direct comparison is crucial for determining whether these alternative methods can reliably replace or complement serum bilirubin assays in routine clinical practice, particularly in settings where rapid healthcare decision-making is critical. Our study aims to cross-compare both TcB and BGA against SBR to evaluate their relative accuracy and determine their clinical utility as screening tools for neonatal jaundice.

## 2. Materials and Methods

### 2.1. Patients

This retrospective observational study included all neonates at Northern Health who underwent clinically indicated bilirubin testing between November 2023 and October 2024. Gestational ages ranged from 27 weeks + 4 days to 42 weeks + 5 days, determined via either last menstrual period or first trimester ultrasound. Inclusion criteria were as follows: (1) neonates assessed as clinically jaundiced and requiring bilirubin testing, (2) neonates who subsequently received all three bilirubin measurements (SBR, BGA, and TcB), and (3) neonates with simultaneous TcB measurements at both the mid-sternal and forehead sites. Neonates were excluded if they had major congenital anomalies, required neonatal transfer to a tertiary centre, or did not receive all three specified bilirubin measurements concurrently.

Maternal and neonatal clinical data were collected retrospectively through a chart review. Variables included gestational age, gender, birth weight, postnatal age, prematurity status, mode of delivery, APGAR score, and ethnicity region. Bilirubin measurement data (SBR, BGA, TcB) and their corresponding time points were also recorded. Additional clinically relevant risk factors and potential confounders were also collected, including respiratory distress, neonatal hypoglycaemia, early presumed onset of sepsis, haemoglobin levels, and type of jaundice treatment. To assess the impact of skin pigmentation on TcB accuracy, Fitzpatrick skin types were inferred from ethnicity region, with types I–II classified as “light” and III–VI as “dark”.

This study was conducted in accordance with the ethical principles of the Declaration of Helsinki and approved by the Northern Health Research Development and Governance Unit (QA reference number: 50.2024). Informed consent was waived due to the study’s retrospective nature. Study data were collected and managed using REDCap electronic data capture tools hosted at Northern Health, Melbourne 3076, Australia (version 12.5.15) and stored on internal servers [18].

### 2.2. Bilirubin Measurement Principles

Bilirubin levels were measured using three methods: total serum bilirubin (SBR) [19], blood gas analyser (BGA), and transcutaneous bilirubinometry (TcB).

SBR values were obtained via an automated clinical chemistry assay in which bilirubin couples with a diazo reagent in the presence of a surfactant to form azobilirubin. The increase in absorbance at 548 nm due to azobilirubin is directly proportional to the total bilirubin concentration.

BGA values were obtained using the Radiometer ABL90 blood gas analyser (Radiometer Medical ApS, Brønshøj, Denmark) [20], which uses multi-wavelength spectrophotometric analysis to quantify total bilirubin from small-volume whole-blood samples [8]. The analyser uses direct optical absorbance to estimate bilirubin concentration and reports values as plasma-equivalent bilirubin.

TcB measurements were obtained using the Dräger JM-10521 (Drägerwerk AG & Co. KGaA, Lübeck, Germany), a non-invasive skin reflectance spectrophotometer [21]. The device emits multiple wavelengths of light into the skin and analyses the absorption and reflection of light by subcutaneous bilirubin to estimate bilirubin concentration. Measurements were taken at both the forehead and mid-sternal regions, and the average value was recorded.

### 2.3. Statistical Analysis

Descriptive statistics were used to summarise the study population. Normality of continuous variables was assessed using the Shapiro–Wilks test. Normally distributed data were summarised with means and standard deviations; non-normally distributed data were reported as medians with interquartile ranges (25th–75th percentiles). Categorical variables were reported as counts and percentages.

Passing–Bablok regression [22], Bland–Altman analysis [23], and Pearson’s correlation [24], were used to examine the differences between SBR-, BGA-, and TcB-derived bilirubin levels. These analyses were also stratified by phototherapy status (pre- vs. post-treatment) to evaluate the effect of phototherapy on measurement accuracy.

The predictive performance of BGA and TcB for identifying neonates requiring phototherapy was assessed by applying locally derived phototherapy threshold charts, which categorises risk based on postnatal age [4]. Infants requiring phototherapy were defined as having hyperbilirubinaemia. The sensitivity, specificity, accuracy, negative predictive value, positive predictive value (PPV), false discovery rate (FDR), and diagnostic odds ratio were calculated using SBR values as the reference standard. The 95th percentile threshold was used, which is consistent with local phototherapy initiation guidelines [25]. All clinical comparisons were based on each neonate’s first recorded sample, prior to phototherapy or exchange transfusion.

To assess the potential confounding effect of haemoglobin on measurement accuracy, subgroup analyses were conducted on neonates with available haemoglobin data. A generalised additive model was used to explore non-linear relationships between haemoglobin levels and the difference between BGA vs. SBR and TcB vs. SBR.

To examine whether skin pigmentation influenced TcB performance, analyses were stratified by Fitzpatrick skin type (light: I–II vs. dark: III–VI). Passing–Bablok and Bland–Altman analyses were conducted separately by group to evaluate potential systematic differences. Additionally, predictive accuracy metrics were compared between skin types. This analysis was repeated using BGA as a comparator.

Lastly, for neonates requiring sequential bilirubin monitoring during jaundice management, paired SBR and BGA measurements were used to assess agreement in tracking bilirubin change over time. The change in bilirubin concentration (∆ bilirubin, μmol/L) was calculated for each consecutive pair of measurements within individual neonates. For neonates with multiple follow-up measurements, all possible sequential pairs were included in the analysis (e.g., for three measurements: initial to first repeat and first repeat to second repeat). Statistical comparison of SBR and BGA ∆ bilirubin distributions was performed using paired *t*-test for means and F-test for variances. Pearson’s correlation coefficient was also calculated to assess the strength of association between methods. Agreement between methods was further evaluated by calculating the difference in ∆ bilirubin values (∆ bilirubin SBR − ∆ bilirubin BGA) for each sequential pair, providing a measure of concordance in tracking bilirubin trends.

## 3. Results

### 3.1. Demographics

A total of 333 measurements were collected from 221 neonates between November 2023 and October 2024. Of these, 113 (51.1%) neonates had multiple measurements during their admission. A total of 181 (54.4%) bilirubin measurements were performed either on infants who did not go on to have phototherapy, or as the initial threshold measurement prior to phototherapy. In total, 99 (44.8%) neonates were diagnosed with serum hyperbilirubinaemia. Nearly half of the cohort were premature, and all treated neonates received phototherapy without requiring exchange transfusion. Table 1 summarises key demographics and clinical characteristics, including gestational age, birth weight, mode of delivery, and clinical risk factors.

### 3.2. Association Between Bilirubin Measurements

**Figure 1:**
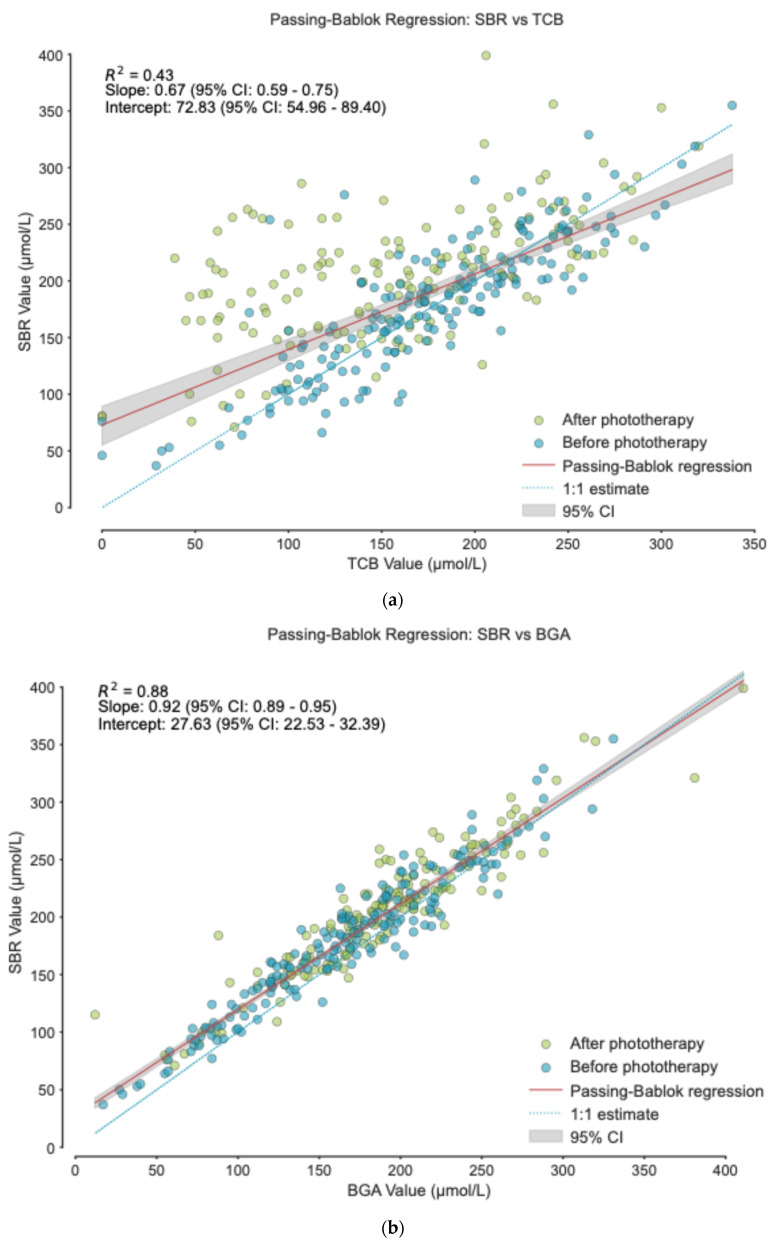
Passing–Bablok regressions: (**a**) SBR vs. TcB; (**b**) SBR vs. BGA.

Passing–Bablok regression (Figure 1) showed stronger agreement between SBR and BGA than between SBR and TcB. Both BGA and TcB showed systematic and proportional differences when compared with SBR, though these were more pronounced for TcB. TcB tended to overestimate bilirubin at low concentrations and underestimate at high concentrations (Figure 1a), while BGA showed only mild overestimation below 300 μmol/L and close agreement above this threshold (Figure 1b).

**Figure 2:**
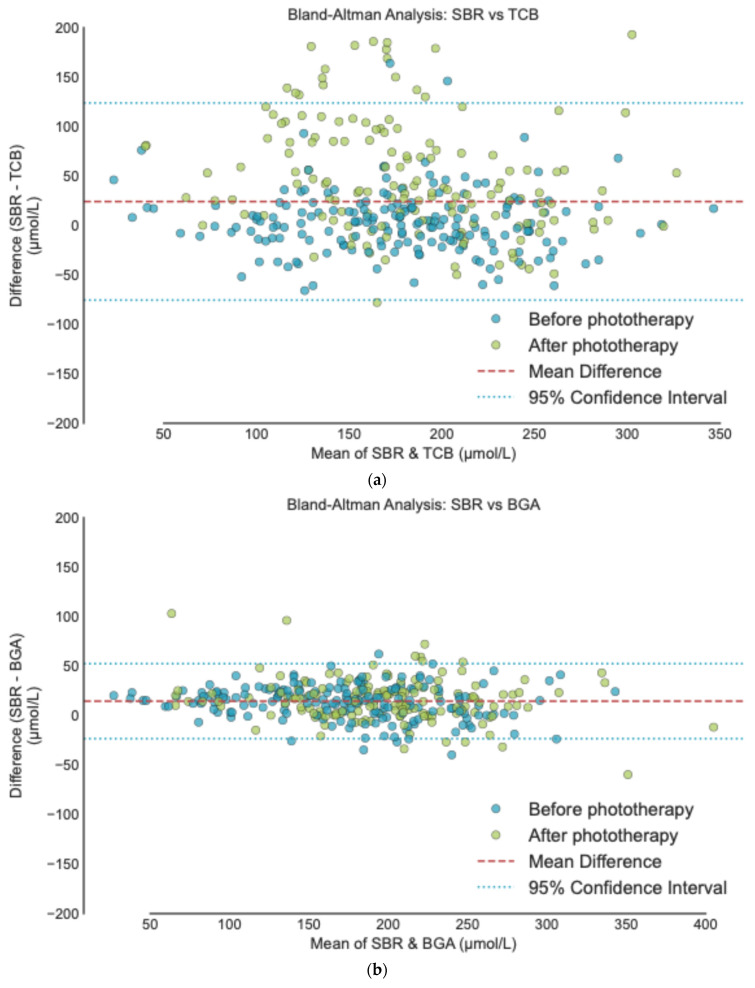
Bland–Altman analysis: (**a**) SBR vs. TcB. Mean difference = 24.1 (95% CI: −75.6–123.8). (**b**) SBR vs. BGA. Mean difference = 14.3 (95% CI: −23.6–52.3). Units are µmol/L.

Bland–Altman plots (Figure 2) revealed a narrower 95% limit of agreement (LoA) between SBR and BGA (Figure 2a) compared to SBR and TcB (Figure 2b). BGA’s LoA was approximately 2.9 times narrower than TcB. While SBR-BGA residuals were symmetrically distributed, SBR-TcB showed an asymmetrical pattern, indicating a tendency to underestimate the true bilirubin levels.

Stratified analysis by phototherapy status (Supplementary Figures S1 and S2) revealed a close agreement between SBR and BGA pre- and post-phototherapy, while the agreement between SBR and TcB dropped markedly. Similarly, Bland–Altman plots showed stable LoA for BGA pre- and post-phototherapy, but a noticeable increase in LoA for TcB after phototherapy.

Lastly, Spearman correlation coefficients mirrored the above findings (Table S1). Spearman correlation coefficients pre- and post-phototherapy were high and almost identical for SBR vs. BGA and were additionally significantly higher compared to SBR vs. TcB. SBR vs. TcB coefficients also declined significantly following phototherapy, providing further statistical evidence that phototherapy leads to a degradation in TcB performance.

### 3.3. Predictive Performance of BGA and TcB

Table 2 presents the performance metrics of BGA and TcB in identifying neonates requiring phototherapy according to Bhutani nomogram thresholds (>95th percentile). Both modalities demonstrated high specificity and comparable negative predictive values. However, BGA showed superior positive predictive value and higher diagnostic odds ratio with a lower false discovery rate compared to TcB. Overall accuracy was also slightly higher for BGA.

### 3.4. Effect of Haemoglobin on BGA Accuracy

**Figure 3:**
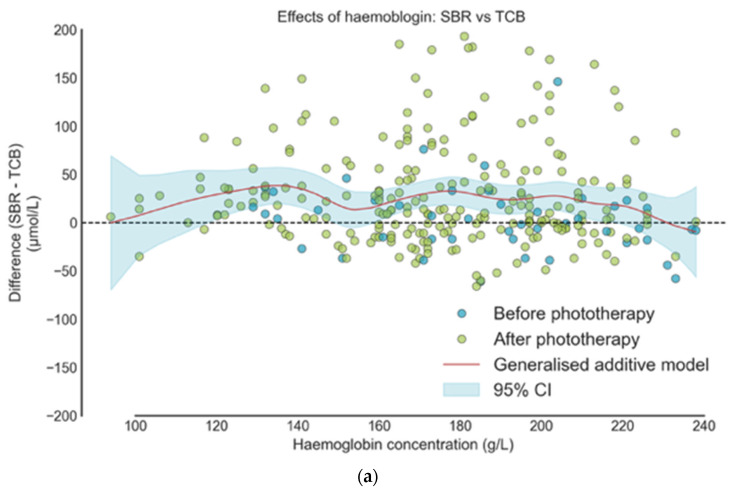

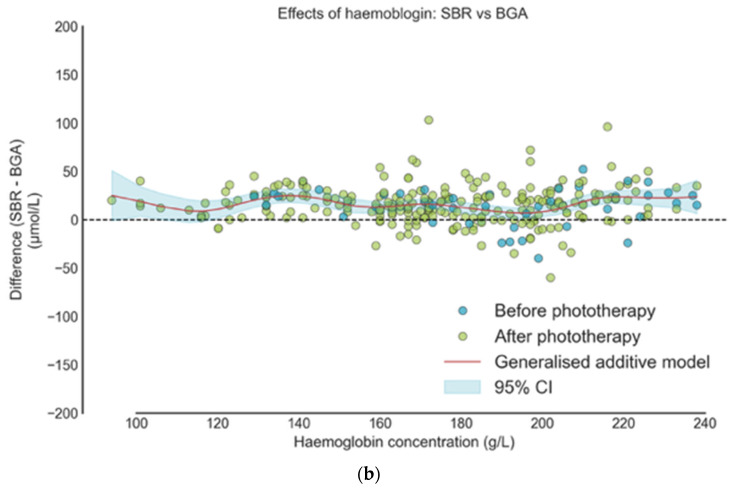
Relationship between haemoglobin and jaundice measures: (**a**) SBR vs. TcB; (**b**) SBR vs. BGA.

Figure 3 shows the relationship between haemoglobin concentration and the difference between BGA and TcB measurements relative to SBR, using generalised additive models. For BGA (Figure 3b), the curve remains flat with narrow confidence intervals, indicating no significant effect of haemoglobin on measurement accuracy. For TcB (Figure 3a), there is minor non-linear variation at lower haemoglobin levels, but the effect is small and not likely to be clinically significant. Overall, haemoglobin concentration does not appear to meaningfully impact the accuracy of either BGA or TcB, and increases in haemoglobin concentration are not significantly associated with deviations in BGA or TcB performance.

### 3.5. Effect of Skin Colour on TcB Accuracy

**Figure 4:**
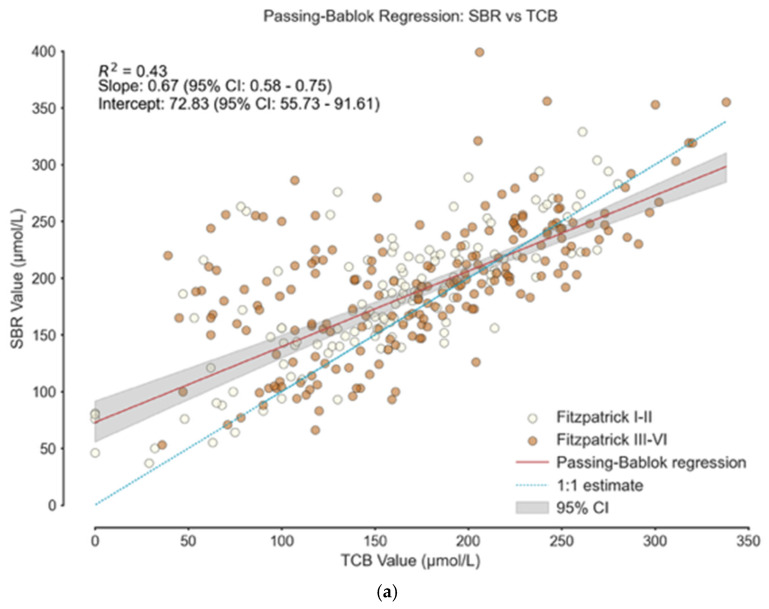

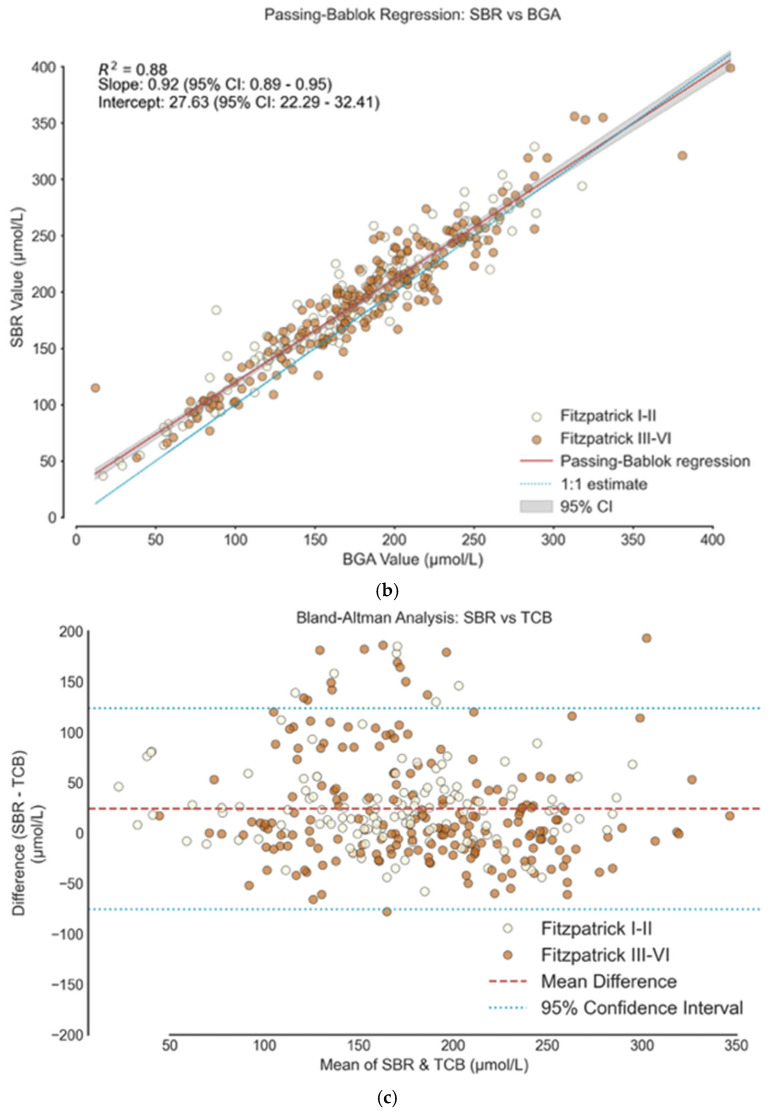

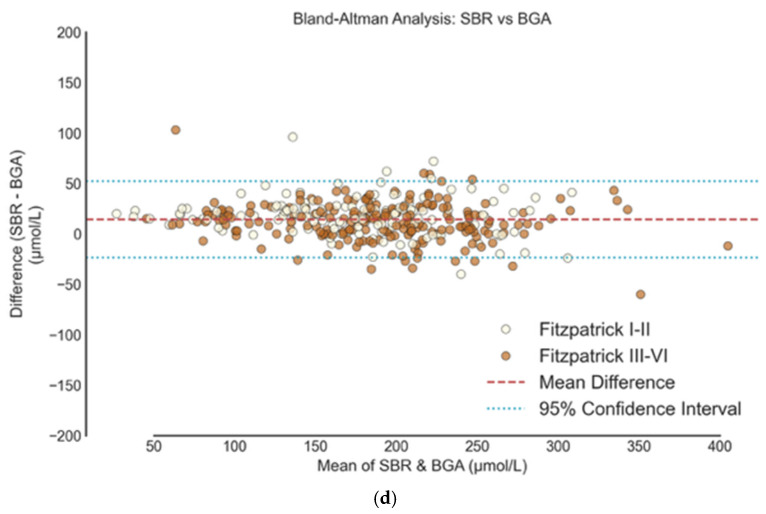
Passing–Bablok regression and Bland–Altman analysis—grouped by Fitzpatrick classifications: (**a**) Passing–Bablok regression of SBR vs. TcB; (**b**) Passing–Bablok regression of SBR vs. BGA; (**c**) Bland–Altman analysis of SBR vs. TcB; (**d**) Bland–Altman analysis of SBR vs. BGA. For (**c**): Fitzpatrick I–II: mean = 26.74, 95% CI = [−56.43, 109.91]; Fitzpatrick III–VI: mean = 22.37, 95% CI = [−86.77, 131.51]. For (**d**): Fitzpatrick I–II: mean = 18.13, 95% CI = [−18.80, 55.06]; Fitzpatrick III–VI: mean = 11.86, 95% CI = [−26.02, 49.73].

Figure 4 shows the distribution of Fitzpatrick skin types across TcB measurements. No distinct clustering or separation by skin type was observed in the Passing–Bablok or Bland–Altman analysis compared to BGA measurements. However, predictive performance varied by Fitzpatrick group (Table S2). For TcB, neonates with darker skin (Fitzpatrick III–VI) had a higher FDR and lower PPV than those with lighter skin, suggesting TcB may overestimate bilirubin levels in darker-skinned neonates. These differences were not observed in BGA performance.

### 3.6. Comparison of SBR and BGA Measurements for Sequential Tracking Performance

A total of 175 sequential bilirubin measurements were performed during the study period, with individual infants having 1–3 repeat measurements. Statistical analysis revealed no significant differences between SBR and BGA ∆ bilirubin distributions (paired *t*-test: *p* = 0.642; F-test for variances: *p* = 0.498). The methods demonstrated strong correlation in ∆ bilirubin (Pearson r = 0.971).

Analysis of individual measurement differences (∆ bilirubin SBR − ∆ bilirubin BGA) showed strong agreement between the two methods. The median and mode differences were both zero, and approximately 50% of sequential measurements demonstrated agreement within 10 μmol/L. Box plot analysis of the comparison is presented in (Figure S3).

## 4. Discussion

In this retrospective cohort study of 333 bilirubin measures from 221 neonates, we identified distinct differences in accuracy and clinical performance of blood gas analyser and transcutaneous bilirubinometry when compared with SBR. Our findings, derived from Passing–Bablok Regression, Bland–Altman Analysis, and Spearman Correlation, illustrate that BGA shows stronger agreement with SBR than TcB. TcB performance also declined significantly post-phototherapy, while BGA remained essentially the same.

Both BGA and TcB demonstrated high specificity but low sensitivity in identifying clinically significant hyperbilirubinaemia requiring phototherapy, based on our phototherapy hyperbilirubinaemia threshold chart of 95%. Notably, TcB had lower positive predictive value among neonates with darker skin (Fitzpatrick III–VI), suggesting a tendency to overestimate bilirubin levels in this group. Our generalised additive model analysis showed no significant trends between haemoglobin levels and the difference between BGA or TcB measurements and SBR.

Our findings regarding BGA-SBR agreement are consistent with prior studies, though the correlation in our data was slightly stronger [10,13,14]. Unlike previous reports that noted performance degradation at higher bilirubin levels [10], our results showed consistent agreement across the full range of values. Additionally, we also found that haemoglobin concentration did not significantly affect the BGA-SBR agreement, contrasting previous study findings [14].

In contrast, our TcB findings diverge from some prior literature. Compared to earlier studies, TcB in our cohort showed lower correlation with SBR and reduced sensitivity, though specificity remained high [9,11,12]. These discrepancies may be due to greater ethnic and skin tone diversity in our population. Prior studies often examined more homogeneous populations [9], while our study included a broader range of Fitzpatrick classifications. Our findings are supported by literature noting TcB variability across skin tones and phototherapy status [11,15,16]. To our knowledge, this is the first study comparing BGA and TcB concurrently in the same cohort, offering unique insight into their relative performance.

Mechanistically, BGA directly measures bilirubin via spectrophotometric analysis of haemolysed blood and applies algorithms to report plasma-equivalent bilirubin concentration, which likely explains its close alignment with SBR [13]. In contrast, TcB devices estimate bilirubin concentration via skin reflectance and are more prone to interference from factors such as skin pigmentation and phototherapy exposure [15]. We observed TcB underestimation at higher bilirubin levels, likely due to optical signal saturation, as previously described [26]. Phototherapy may further reduce TcB readings by “bleaching” the skin or redistributing bilirubin into deeper tissues, a phenomenon supported both by our data and prior work [27].

As foetal haemoglobin (HbF) is the predominant form of haemoglobin in newborns, we indirectly observed the impact of HbF concentration on bilirubin measurement accuracy. Haemoglobin concentration potentially interferes with spectral data due to overlap with bilirubin. Notably, BGA showed lower susceptibility to HbF interference compared to TcB. The Radiometer ABL-90 analyser in our study incorporates spectral data corrections specifically for HbF, enabling accurate estimation of plasma-equivalent bilirubin concentration despite varying HbF concentrations [20]. In contrast, TcB measurements lack this capability to directly correct for HbF interference, with algorithms which convert TcB measurements to bilirubin values relying on estimates of haemoglobin which decrease by 10% in the first week of life [28]. Finally, haemoglobin does not directly interfere with the diazo SBR method in non-haemolysed specimens. However, oxyhaemoglobin present in plasma due to haemolysis results in an underestimation of bilirubin concentration [29].

Management of neonatal hyperbilirubinaemia relies on two critical clinical functions: identification of neonates requiring phototherapy intervention, and subsequent monitoring of bilirubin trends to assess treatment response and confirm jaundice resolution. The findings of this study have important healthcare implications for both aspects of clinical care.

When compared with the reference standard laboratory diazo bilirubin methodology, our findings support the use of BGA as a reliable screening tool for neonatal hyperbilirubinaemia, especially in high-acuity settings. Although TcB demonstrated lower overall accuracy, it remains valuable as a non-invasive screening method and aligns with local guidelines that recommend TcB for initial jaundice screening, with SBR confirmation required when values exceed predetermined treatment thresholds.

Given the low sensitivity of both alternative methods compared to SBR, clinicians should consider using conservative cutoffs when assessing the need for phototherapy. The high specificity and negative predictive values of both methods support their role in confidently excluding the need for treatment, thereby reducing unnecessary blood sampling. This is particularly relevant in low-risk neonates or clinical settings where venepuncture should be minimised. While measurement of bilirubin via BGA does not eliminate invasive blood sampling, the blood volume reduction can be significant as the 65–100 µL required for BGA is five times less than what is required for SBR. For premature infants, who are often sampled multiple times, such a volume reduction is clinically important and provides an added benefit when the infant is already undergoing multiple other BGA analyses. Additionally, the promptness of the result facilitates a real-time clinical response. However, caution is advised when considering BGA as a replacement for SBR in ambiguous or high-risk cases. Such cases may include where there is early or prolonged jaundice, where the jaundice is inconsistent with the clinical state, clinical instability, haemolytic disease, family history of hyperbilirubinaemia, birth trauma, and conjugated hyperbilirubinaemia.

BGA showed superior discriminatory performance with a diagnostic odds ratio of 47.6, approximately 2.5-fold higher than TcB (19.3). This finding suggests that BGA is markedly more effective in distinguishing between neonates who do and do not require phototherapy, supporting its use as an alternative to SBR in resource-limited or time-sensitive contexts where rapid, reliable results are essential.

The variability observed in TcB accuracy has practical implications. While TcB offers a valuable non-invasive screening option, its tendency to overestimate bilirubin in darker-skinned neonates and underestimate values post-phototherapy means it should not be used in isolation for making treatment decisions. Positive TcB findings should be confirmed with SBR to prevent misclassification and overtreatment, consistent with recommendations from prior reviews [15,16].

This study employed a comparative methodology consistent with previous research by evaluating BGA and TcB measurements against the established laboratory-based SBR reference standard. This approach reflects the longstanding clinical need for alternative methodologies offering reduced analytical interference susceptibility, less invasive sampling requirements, and point-of-care accessibility to minimise processing delays. The interpretation of sensitivity findings requires acknowledgement of inherent limitations in the reference methodology. The diazo-based SBR method is subject to well-documented analytical interferences from haemolysis, lipaemia, and paraproteins, which may influence the accuracy of comparative assessments [30]. Additionally, the widely utilised phototherapy hyperbilirubinaemia threshold charts are arguably predominantly derived using diazo methodology data, creating potential methodological interdependency in the evaluation of alternative approaches.

Despite these methodological considerations, our sequential monitoring analysis shows the clinical utility of BGA which showed close agreement via Passing–Bablok regression and Spearman correlation with SBR both pre- and post-phototherapy. Comparison of SBR and BGA ∆ bilirubin distributions revealed no statistical differences with high correlation (r = 0.971). Importantly, analysis of sequential measurement agreement demonstrated medial and mode differences of zero, with 50% of sequential measurements achieving agreement within 10 μmol/L. These findings support clinical confidence in using BGA bilirubin measurements for tracking jaundice resolution, offering practical advantages of minimal sample volume requirements and real-time point-of-care results.

This study has limitations. First, Fitzpatrick classification was retrospectively inferred from maternal ethnicity rather than direct assessment, potentially introducing misclassification bias. Second, the single-centre design using specific devices and protocols may limit generalisability to other clinical settings. It should be noted that the intent of this retrospective study was not to promote BGA as a replacement for TcB or SBR in clinical decision-making. Our objective was to perform a direct comparison of BGA-derived bilirubin and TcB measurements against serum SBR within the same cohort, to provide a previously unavailable analytical perspective with potential new insights—while avoiding between-study variability that arises when different modalities are assessed in separate populations.

Future investigations should aim to include larger, multi-centre cohorts with balanced pre- and post-phototherapy data, direct assessment of skin pigmentation, and standardised device calibration protocols. Such efforts will help refine bilirubin screening protocols and ensure accurate interpretation across diverse neonatal populations.

## 5. Conclusions

The healthcare implications of this study are that BGA represents a more reliable alternative to SBR than TcB for neonatal hyperbilirubinaemia assessment, particularly in time-critical or resource-limited clinical settings. BGA’s superior diagnostic performance, consistent accuracy across different clinical conditions, and effectiveness in sequential monitoring support its implementation as both a screening tool and for tracking jaundice resolution. While TcB retains value as a non-invasive screening method, its limitations post-phototherapy and reduced accuracy in neonates with darker skin necessitate confirmatory SBR testing in positive cases. These findings advocate for selective and context-aware implementation of BGA and TcB to optimise neonatal hyperbilirubinaemia management while minimising unnecessary interventions and invasive procedures.

## Figures and Tables

**Table 1:** Demographic and clinical risk factor summary.

Demographic	Values
Gestational age, weeks (median, IQR)	37.1 [35.0, 39.0]
Postnatal age, days (mean, SD)	3.5 (2.9)
Male sex (n, %)	135 (61.1)
Birth weight, gram (mean, SD)	2803.8 (828.9)
Fitzpatrick ≥ III (n, %)	139 (62.9)
Prematurity (n, %)	102 (46.2)
**Mode of delivery**	
Vaginal (n, %)	114 (51.6%)
Caesarean (n, %)	107 (48.4)
**Jaundice treatment**	
None (n, %)	122 (55.2)
Phototherapy (n, %)	99 (44.8)
APGAR at 5 min < 7 (n, %)	8 (3.6)
Respiratory distress (n, %)	73 (33.0)
Hypoglycaemia (n, %)	60 (27.2)
Prophylactic antibiotics (n, %)	99 (44.8)
Haemoglobin (n = 186)	178.5 [151.0, 199.0]

**Table 2:** Performance of measures in deciding phototherapy.

	BGA	TcB
True Positives (n)	22	22
False Positives (n)	5	12
True Negatives (n)	281	274
False Negatives (n)	26	26
Sensitivity (%)	45.8%	45.8%
Specificity (%)	98.3%	95.8%
Accuracy (%)	90.7%	88.6%
Positive Predictive Value (%)	81.5%	64.7%
Negative Predictive Value (%)	91.5%	91.3%
False Discovery Rate (%)	18.5%	35.3%
Diagnostic odds ratio [95% CI]	47.6 [16.6, 135.1]	19.3 [8.6, 43.4]

## Data Availability

The data that support the findings of this study are not publicly available due to the fact that they containing information that could compromise the privacy of research participants but are available from the corresponding author upon reasonable request.

## Supplementary Materials

The following supporting information can be downloaded at https://www.mdpi.com/article/10.3390/healthcare14030370/s1, Figure S1. Passing-Bablok regression before & after phototherapy: (a) SBR vs. TcB, before phototherapy; (b) SBR vs. TcB, after phototherapy; (c) SBR vs. BGA, before phototherapy; (d) SBR vs. BGA, after phototherapy. Figure S2. Bland-Altman analysis before & after phototherapy: (a) SBR vs. TcB, before phototherapy. Mean difference = 3.76, 95% CI [−60.28, 67.79] (b) SBR vs. TcB, after phototherapy. Mean difference = 48.42, 95% CI [−64.76, 161.61] (c) SBR vs. BGA, before phototherapy. Mean difference = 13.20, 95% CI [−20.58, 46.97] (d) SBR vs. BGA, after phototherapy. Mean difference = 15.66, 95% CI [−26.68, 58.00]. Figure S3. Box plot of agreement of ∆ bilirubin SBR-∆ bilirubin BGA for sequential bilirubin tracking. Table S1. Spearman correlation coefficients. Table S2. Performance measures in deciding phototherapy—grouped by Fitzpatrick classifications.

## Abbreviations

The following abbreviations are used in this manuscript:

APGAR	Appearance, Pulse, Grimace, Activity, Respiration score
BGA	Blood Gas Analyser
FDR	False Discovery Rate
HbF	Foetal Haemoglobin
LoA	Limit of Agreement
PPV	Positive Predictive Value
SBR	Serum Bilirubin
TcB	Transcutaneous Bilirubinometry
