# Supplementary material for: Point-of-Care Bilirubin Testing in Neonates: Comparative Performance of Blood Gas Analysis and Transcutaneous Bilirubinometry
Source: Healthcare (Basel). 2026 Feb 1;14(3):370. doi: 10.3390/healthcare14030370 (PMC12897715; doi:10.3390/healthcare14030370)
Supplement: Supplementary file 1 [file healthcare-14-00370-s001.zip › healthcare-4095994-supplementary.pdf]

Supplementary Material

(a)

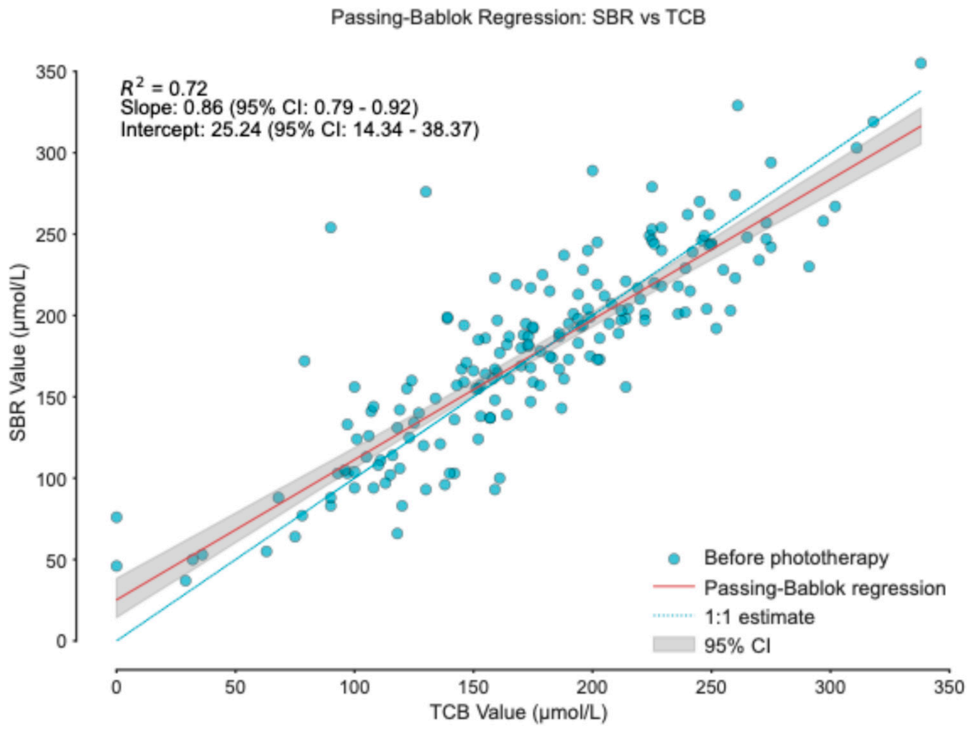

(b)

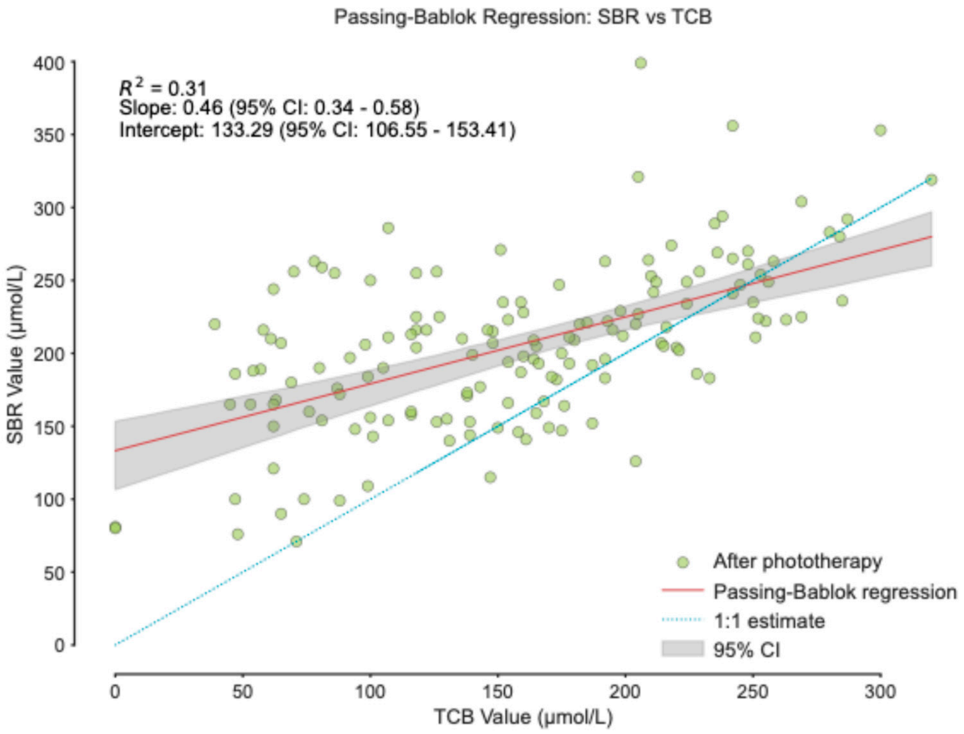

(c)

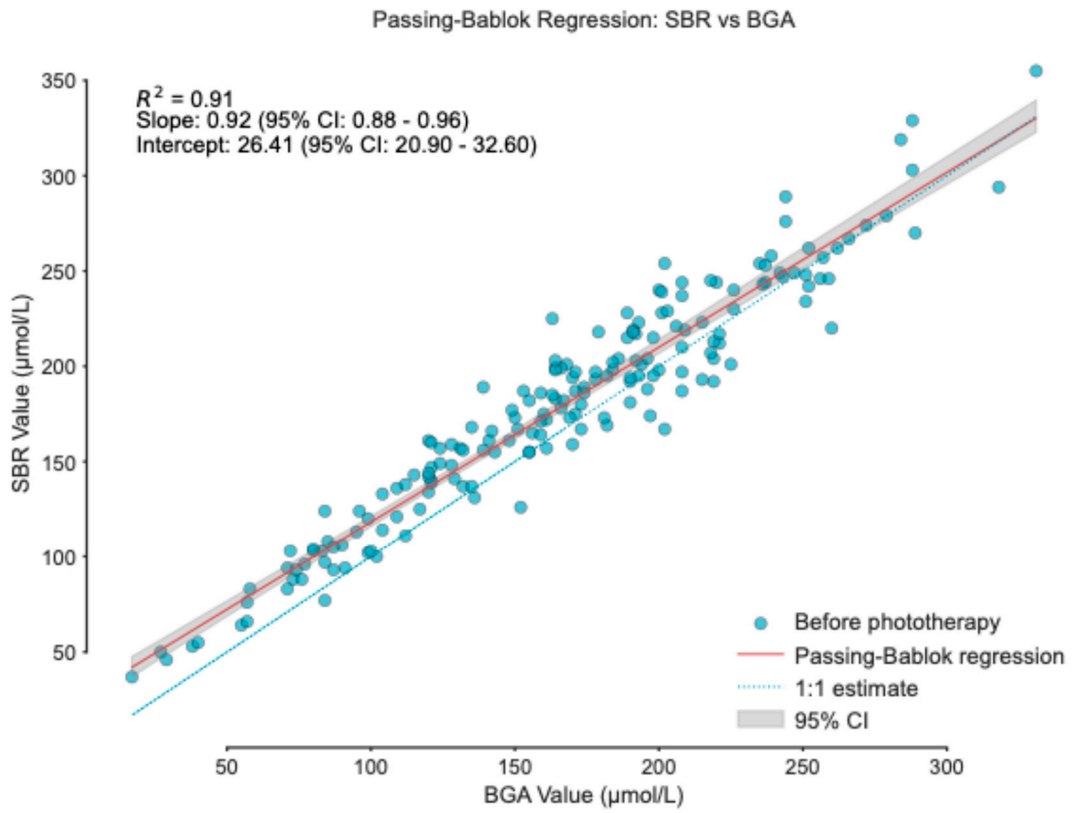

(d)

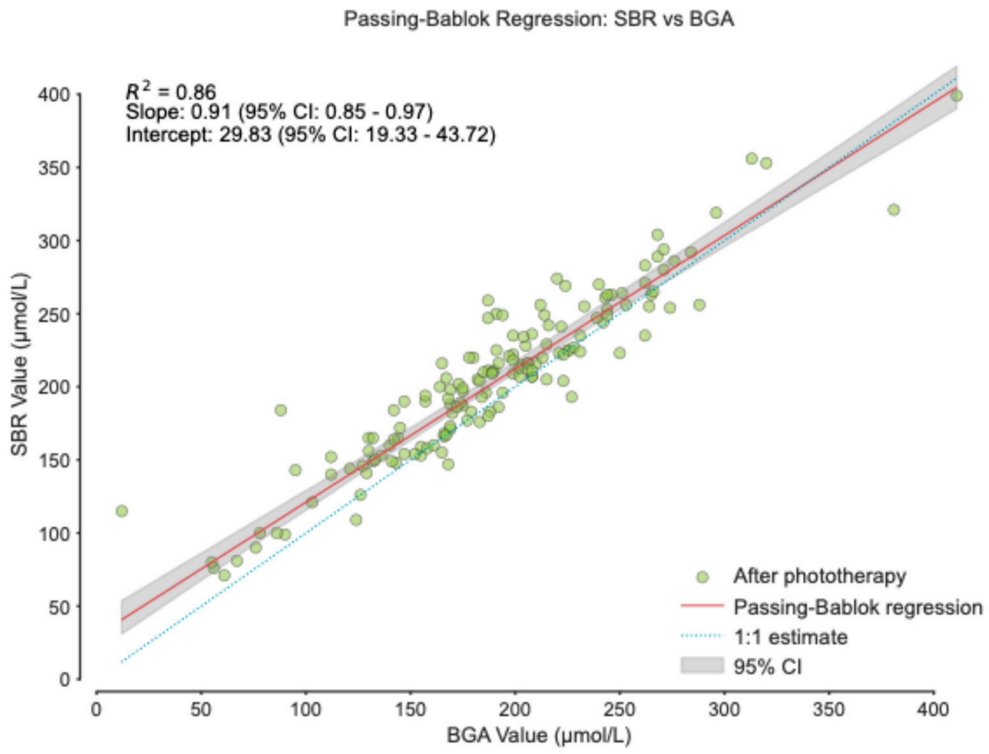

Figure S1. Passing-Bablok regression before & after phototherapy: (**a**) SBR vs. TcB, before phototherapy; (**b**) SBR vs. TcB, after phototherapy; (**c**) SBR vs. BGA, before phototherapy; (**d**) SBR vs. BGA, after phototherapy

(a)

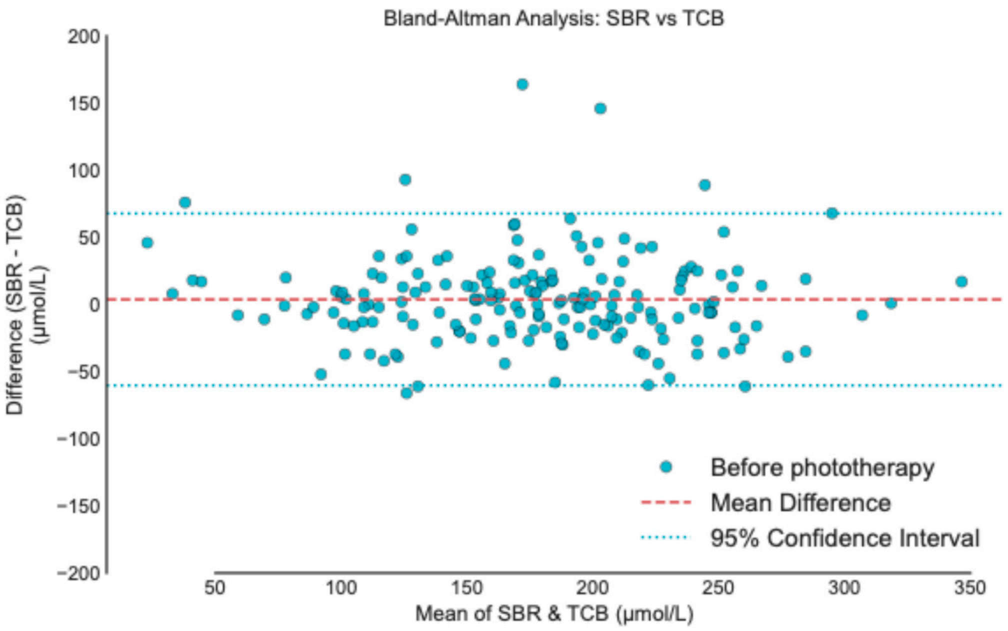

Bland-Altman Analysis: SBR vs TCB
Difference (SBR - TCB) (μmol/L)
Mean of SBR & TCB (μmol/L)
Before phototherapy
Mean Difference
95% Confidence Interval

(b)

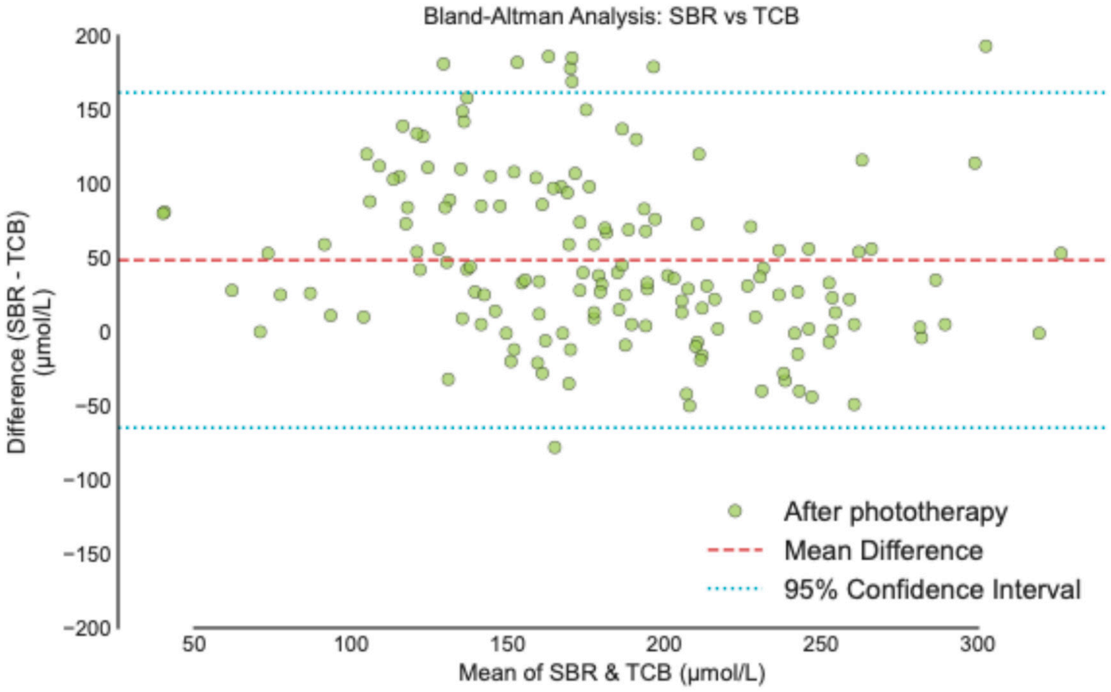

Bland-Altman Analysis: SBR vs TCB
Difference (SBR - TCB) (μmol/L)
Mean of SBR & TCB (μmol/L)
After phototherapy
Mean Difference
95% Confidence Interval

(c)

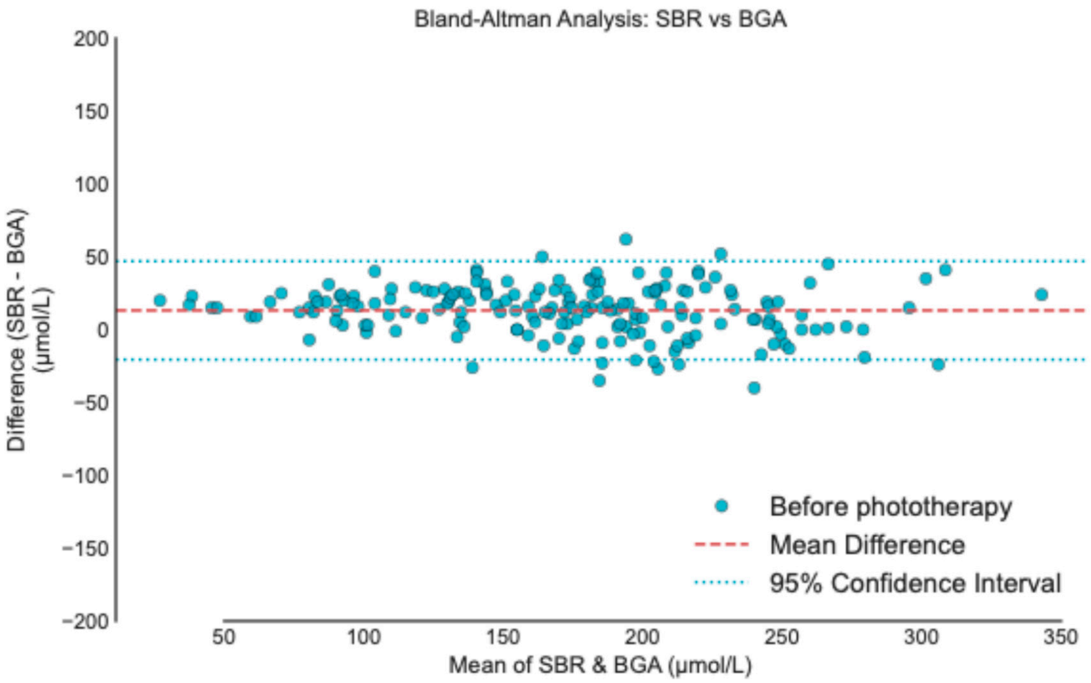

(d)

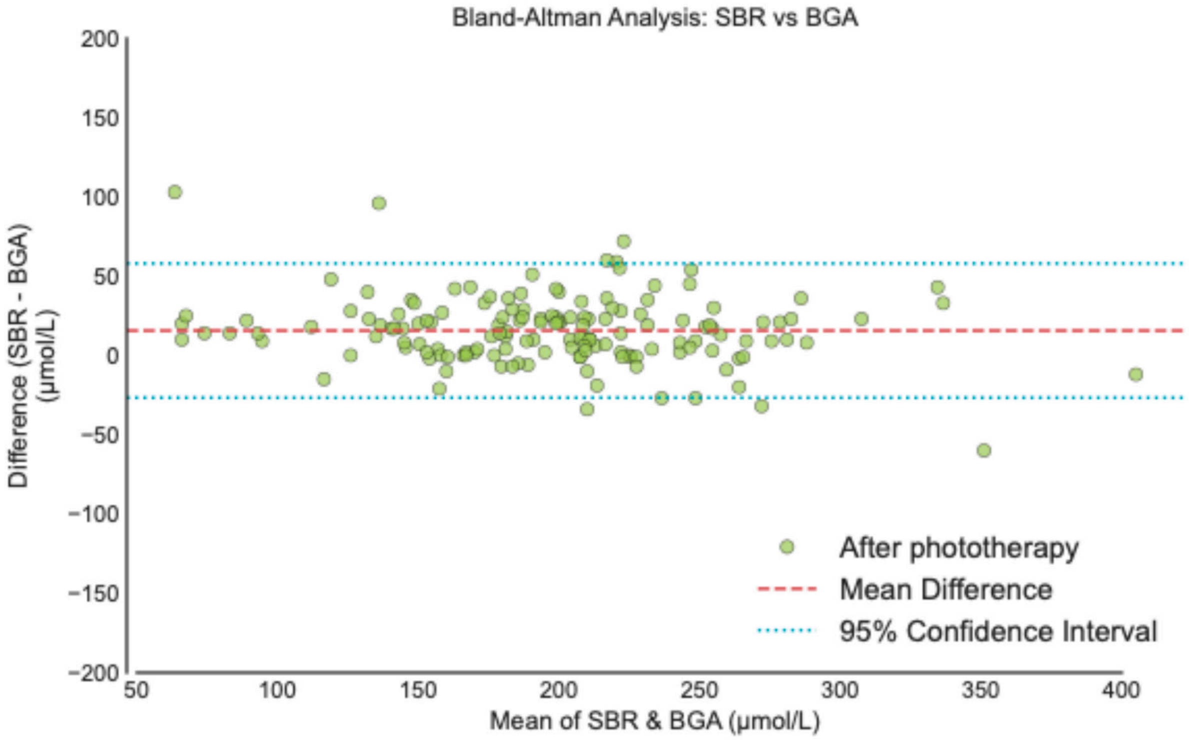

Figure S2. Bland-Altman analysis before & after phototherapy: (**a**) SBR vs. TcB, before phototherapy. Mean difference = 3.76, 95% CI [-60.28, 67.79] (**b**) SBR vs. TcB, after phototherapy. Mean difference = 48.42, 95% CI [-64.76, 161.61] (**c**) SBR vs. BGA, before phototherapy. Mean difference = 13.20, 95% CI [-20.58, 46.97] (**d**) SBR vs. BGA, after phototherapy. Mean difference = 15.66, 95% CI [-26.68, 58.00].

Table S1. Spearman correlation coefficients.

	Spearman correlation	95% CI
	**All**	
SBR vs BGA	0.94	(0.92, 0.96)
SBR vs TcB	0.66	(0.58, 0.73)
BGA vs TcB	0.67	(0.60, 0.74)
	**Before phototherapy**	
SBR vs BGA	0.95	(0.93, 0.97)
SBR vs TcB	0.85	(0.78, 0.91)
BGA vs TcB	0.84	(0.77, 0.89)
	**After phototherapy**	
SBR vs BGA	0.93	(0.89, 0.95)
SBR vs TcB	0.56	(0.43, 0.68)
BGA vs TcB	0.59	(0.46, 0.71)

Table S2. Performance measures in deciding phototherapy – grouped by Fitzpatrick classifications

	Fitzpatrick I - II		Fitzpatrick III - IV	
	**BGA**	**TcB**	**BGA**	**TcB**
True Positives (n)	6	7	16	15
False Positives (n)	1	3	4	9
True Negatives (n)	110	108	171	166
False Negatives (n)	14	13	12	13
Sensitivity (%)	30%	35.0%	57.1%	53.6%
Specificity (%)	99.1%	97.3%	97.7%	94.9%
Accuracy (%)	88.6%	87.8%	92.1%	89.2%
Positive Predictive Value (%)	85.7%	70.0%	80.0%	62.5%
Negative Predictive Value (%)	88.7%	89.3%	93.4%	92.7%
False Discovery Rate (%)	14.3%	30.0%	20.0%	37.5%
Diagnostic Odds Ratio [95% CI]	47.1 [5.3, 420.8]	19.4 [4.5, 84.3]	57.0 [16.5, 197.3]	21.3 [7.82, 57.9]

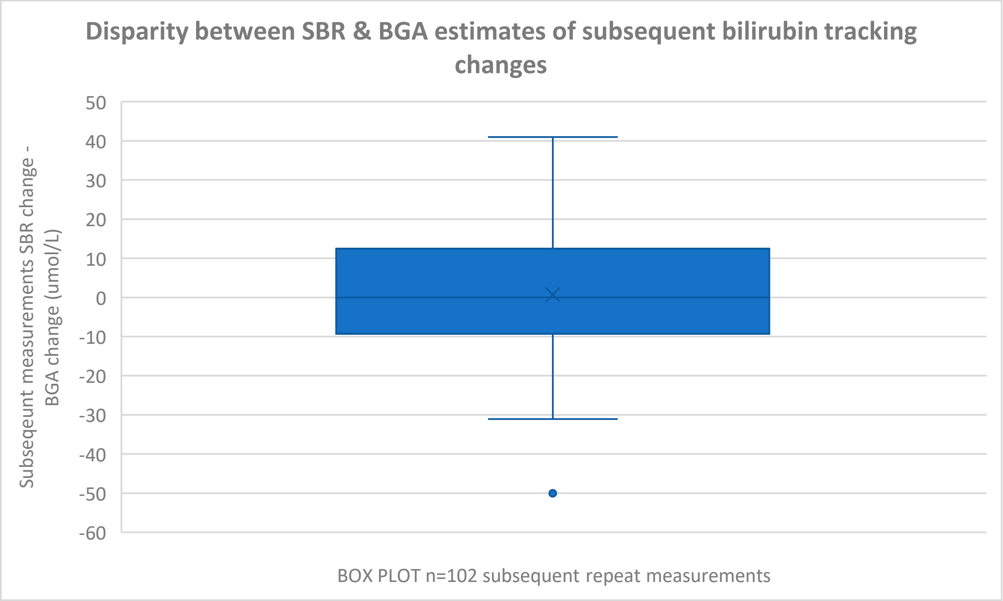

Figure S3. Box plot of agreement of Δ bilirubin SBR - Δ bilirubin BGA for sequential bilirubin tracking.
